# Development of Bioadhesive Transdermal Bupivacaine Gels for Enhanced Local Anesthetic Action

**Published:** 2012

**Authors:** Cheong-Weon Cho, Deok-Bae Kim, Sang-Chul Shin

**Affiliations:** a *College of Pharmacy, Chungnam National University, Daejeon, 305-764, Korea. *; b *College of Medicine, Chosun University, Gwangju 501-759, Korea.*; c *College of Pharmacy, Chonnam National University, Gwangju 500-757, Korea.*

**Keywords:** Bupivacaine, Gels, Penetration enhancer, Transdermal, Local anesthetic action

## Abstract

Topical drug dosage forms such as ointments and creams can be easily removed through wetting, movement and contact. The new bioadhesive formulations with enhanced local anesthetic effects are needed for topical administration. The adhesive capacity of hydroxypropyl methylcellulose (HPMC) was determined by measuring the maximum detachment force and the adhesion work with an auto peeling tester. The release of drug from a HPMC gel was studied according to the drug concentration. Permeation study through the rat skin was performed at 37°C using phosphate buffer solution (pH = 7.4) as a receptor medium. To increase the skin permeation of bupivacaine from the HPMC gels, penetration enhancer such as the saturated and unsaturated fatty acids, the pyrrolidones, the propylene glycol derivatives, the glycerides, and the non-ionic surfactants were incorporated in the bupivacaine-HPMC gels. The local anesthetic effect of the formulated gel preparation was examined using a tail-flick analgesimeter. As the concentration of HPMC increased, the bioadhesive force and viscosity were increased. The rate of drug release was increased with increasing the drug concentration. Among the enhancers used, polyoxyethylene 2-oleyl ether showed the most enhancing effects on drug permeation through the skin. In the rat tail flick test, the area under the efficacy curve of bupivacaine gel containing polyoxyethylene 2-oleyl ether and tetrahydrozoline showed a 2.36-fold increase in anesthetic activity compared to control gel without any additives. The bupivacaine gels containing both penetration enhancer and vasoconstrictor showed enhancement and prolonged efficacy compared to the control gel. To enhance the local anesthetic effects of bupivacaine, the transdermal bupivacaine gel formulation containing penetration enhancer and vasoconstrictor could be developed.

## Introduction

Local anesthetics are widely used in surgical, obstetric and dental patients. They are also used in the control of postoperative pain and in the therapy of chronic pain (1) and can be used for the regional control of major pain. One of these local anesthetic drugs is bupivacaine, which is characterized by its long action and high therapeutic power (2). There is a substantial population with intractable pain that is not responsive to opioids that require non-opioid agents, including local anesthetics (1, 3). In these cases, non-opioid agents including local anesthetics, such as bupivacaine, are used (3, 4). Bupivacaine plays a valuable role in the overall management of surgical and postoperative pain associated with dental care (5, 6). Subcutaneously injected bupivacaine reportedly produces analgesia via a systemic effect (7). Local anesthetics affect a number of biologic processes, including the inhibition of G-protein-coupled receptor signaling, which are potentially important pharmacodynamic actions that are of value in treating pain.

Of many drug delivery systems, percutaneous drug delivery can provide controlled delivery of drugs. However, ointments and creams can be easily removed by wetting, movement, and contact. New bioadhesive formulations are needed to enhance local anesthetic effects.

Hydroxypropyl methyl cellulose (HPMC) is used to control the drug release from several pharmaceutical systems because of its non-toxic nature, easy compression, swelling properties, and accommodation of high levels of drug (8, 9). To formulate bioadhesive gels, we compared the viscosity and bioadhesive forces of HPMC, as well as drug release according to the drug concentration. To increase the skin permeation of bupivacaine from the HPMC gels, enhancers such as saturated and unsaturated fatty acids, pyrrolidones, propylene glycol derivatives, glycerides, and the non-ionic surfactants were incorporated in the bupivacaine-HPMC gels. The local anesthetic effects of the formulated bupivacaine-HPMC gels containing polyoxyethylene 2-oleyl ether and tetrahydrozoline were evaluated using the tail flick anesthetic test.

The objective of this study was to determine the feasibility of the transdermal application related to bupivacaine from a bioadhesive gel formulation through studying *in-vitro* release characteristics and *in-vivo* local anesthetic efficacy.

## Experimental

Bupivacaine hydrochloride was supplied from Hana Pharm. Co. Ltd (Korea). Hydroxypropyl methylcellulose -K100M (MW, 10000) was obtained from DOW chemical Co. Ltd (USA). Tetrahydrozoline was purchased from Sigma Aldrich Inc., (Sigma Aldrich, USA). Lauric acid, oleic acid and caprylic acid were purchased from Tokyo Kasei Kogyo Co., Ltd (Japan). One-methyl-2-pyrrolidone and 2-pyrrolidone were purchased from Acros organics (Acros organics, USA). Myristic acid, linoleic acid, polyoxyethylene-23-lauryl ether (Brij 35), polyoxyethylene-2-oleyl ether (Brij 92), and polyoxyethylene-23-lauryl ether (Brij 72) were purchased from Sigma-Aldrich Co. (Sigma-Aldrich, USA). Oleyl macrogol-6 glycerides, caprylocaproyl mcarogol-8 glycerides, propylene glycol mono caprylate, propylene glycol laurate, and propylene glycol monolaurate were gifts from Gattefose (Gattefose, France). Acetnitrile was HPLC grade from J.T. Baker Inc. (J.T. Baker, USA). All reagents of analytical grade were used without further purification. Double distilled water was used for all studies.

*Measurement of viscosity and bioadhesive strength*

To select the optimum concentration of HPMC-K100M, various concentrations of HPMC gel (1%, 2% and 3%) were prepared, respectively. Each gram of HPMC was dissolved with stirring in hot water to make a 100 g solution.

The viscosity of each gel (100 g) was measured at room temperature with a rotary type viscometer (Haake viscometer, Germany). The sensor system was MV II. The sensor was inserted into a gel sample in the MV II cup and adjusted to a rate of shear of 1.8 (sec^-1^) and sample equilibration took approximately 45 sec. The viscosity of sample was then determined by multiplying the observed reading by the shear rate.

To evaluate the bioadhesive strength of HPMC correctly, rats’ intestines were used (10, 11). The adhesive capacity of HPMC was determined by measuring the maximum detachment force and the adhesion work with an auto peeling tester (C.K. Trading Co. Ltd. Korea). Cyanoacrylate adhesive was used to fix the intestinal mucosa to the upper and lower support. The HPMC gel (1 g) was placed at room temperature on the both supports. Upon contact of the gel-intestine mucosa, a force (contact pressure, 50 gf) was applied for five minutes. The detachment procedure was performed at the speed of 150 mm/min until the complete detachment of the components was achieved. The force required to completely separate the two components was recorded as the adhesion force. The adhesion force was calculated as N (Newton force).

*Preparation of bupivacaine-HPMC gels containing enhancer and vasoconstrictor*

Two grams of hydroxypropyl methylcellulose (HPMC) was dissolved in hot water to make 35 g solution. Bupivacaine (1%) and each enhancer (5%), tetrahydrozoline (0.05%), were added with vigorous stirring to the above HPMC solution and water was added to make a 100 g gel. The appearance of the prepared gel was transparent.

*Release of bupivacaine from the HPMC gels*

The *in-vitro* release of bupivacaine from the HPMC gels was examined using the modified Keshary-Chien cell. The diameter of the cell was 2 cm, providing 3.14 cm^2^ effective constant areas between the membrane and the bulk solution of 20 mL. The flux of bupivacaine from the HPMC gels was determined using phosphate buffer solution (pH = 7.4) as a receptor. The synthetic cellulose membrane (SPECTRA/POR^Ⓡ^ MW 12-14,000, pore size, 0.2 um) was mounted on the receptor compartment of the diffusion cell (12). Five grams of prepared HPMC gels containing bupivacaine was placed in intimate contact with the cellulose membrane and the donor cap was covered with parafilm and clamped. The sampling port was sealed with parafilm to prevent the evaporation of the receptor medium. The receptor solution was maintained at 37°C through a circulating water bath and stirred constantly at 400 rpm. Before the experiment, the system was tested to remove the remaining air bubble in receptor site.

To formulate the appropriate drug concentration, the drug release from the gels was studied at drug concentrations of 0.5, 1, 1.5 and 2% (w/w) at 37°C in a controlled water bath. The total medium from the receptor compartment was withdrawn at predetermined intervals to maintain a sink condition and immediately replaced with the same amount of fresh phosphate buffer solution (pH = 7.4). Each data point represents the average of three determinations.

*HPLC determination of bupivacaine*

Bupivacaine was assayed using HPLC. The HPLC system had a pump (Knauer, DE/K-120, USA.), ultraviolet detector (Waters 484, USA), C_18_ column (250 x 4.6mm, 5um), degaser, and an integrator (D520A, Youngin scientific Co., Ltd., Korea). The mobile phase was composed of a mixture (70:30:0.1, v/v) of water, acetonitrile, and phosphoric acid. A flow rate of 1.0 mL/min yielded an operation pressure of ~1000 psi. The UV detector was operated at the wavelength of 210 nm. Under these conditions, the bupivacaine peak appeared at the retention time of 7.4 min. The calibration curves of bupivacaine were linear within the range of 2-500 ng/mL. The intra- and inter-day coefficients of variation for bupivacaine were less than 1.5%.

*Permeation of bupivacaine from the HPMC gels through the rat skin*

A healthy male rat (Sprague Dawley rat strain) with 7-8 weeks old (270-300 g), was sacrificed by cervical dislocation. The hair of abdominal area was carefully removed with an electric clipper. A square section of the abdominal skin was excised. After the incision, the adhering fat and other visceral debris in the skin were carefully removed from the undersurface with tweezers. The excised skin was used immediately.

The freshly excised full-thickness skin sample was mounted on the receptor site of the diffusion cell with the stratum corneum side facing upwards into the donor compartment and the dermal side facing downwards into the receptor compartment.

The *in-vitro* permeation of bupivacaine from the HPMC gels was examined using the modified Keshary-Chien cell for 120 min. The diameter of the cell was 2 cm, providing 3.14 cm^2^ effective constant areas and the volume of the diffusion cell was 20 mL. The flux of bupivacaine from the HPMC gels was determined using phosphate buffer solution (pH = 7.4) as a receptor. Appropriate amount of gels (1 g) was placed on the stratum corneum side and covered with round glass plate and clamped. Receptor medium was phosphate buffer solution (pH = 7.4) to achieve the sink condition and maintained 37°C through a circulating water bath. Total samples (20 mL) were withdrawn at predetermined intervals (every 30 min) and immediately replaced with an equal volume of fresh medium. Permeation quantities of bupivacaine were analyzed using HPLC at 210 nm. Each data point represents the average of three determinations.

The cumulative amount of bupivacaine through the rat skin was plotted against the time (min). A linear profile was observed for 2 h and the slope of the linear portion of the curve was determined through the linear regression. The effectiveness of penetration enhancers was defined as the enhancement factor (EnF). EnF was calculated using the following equation:

EnF = (flux of HPMC gels containing enhancers) / (flux of the control) 

*Tail flick Test*

Healthy male Sprague-Dawley rats with 7-8 weeks old (270-300 g), were purchased from Daehan Laboratory Animal Research Co. (Choongbuk, Republic of Korea), and were given a commercial rat chow diet (No. 322-7-1, Superfeed Co., Gangwon, Republic of Korea) and tap water ad libitum. The animals were housed (four per cage) in laminar flow cages maintained at 22 ± 2°C and 50-60% relative humidity under a 12:12 h light-dark cycle. The experiments began after allowing at least one week for acclimation. The experiments were performed in accordance with the “Guiding Principles in the Use of Animals in Toxicology” adopted by the Society of Toxicology (USA) in July, 1989, and revised in March, 1999. The Animal Care Committee of Chonnam National University (Gwangju, Republic of Korea) approved the design and conduct of this study (2008-12).

The heat radiant tail flick assay, developed by D’Amour and Smith in the 1940s (13), is a commonly used experimental model for thermo-pain quantification. In this test, a rodent tail is exposed to a light source (radiant heat) and the latency of tail withdrawal from the heat source is recorded and analyzed. Depending on the experimental settings, the tail flick technique can be used to determine the basal nociception level, the analgesic effectiveness of pharmacological agents, and tolerance formation.

The rats were divided into four groups containing three rats each: control gel group, bupivacaine gel group, bupivacaine gel containing enhancer group and bupivacaine gel containing enhancer and vasoconstrictor group.

The statistical significance of the differences between the formulations was tested using the Student’s paired t-test. It was defined to be statistically significant when p < 0.05. Each data point represents the average of three determinations. All values were reported as mean ± standard deviation.

The rat was fixed on a tail-flick-test apparatus (Tail flick anesthetic meter, Harvard Co. Ltd. USA) with the tail of 10 cm from its tip, exposed to heat from a projector lamp. A single control switch simultaneously activated the light and a timer and the timer was stopped automatically when the tail flicked. The time interval between switching on the light to flick of the tail was recorded. A 50 sec cut-off time was used to avoid thermal injury. A dose of 50 mg of drug gel was applied to the root of the tail on the midline. The tail flick anesthetic test was started after the administration and the test was done every 5 min until the duration time fell to control value.

The area under the effective curve from the beginning till the end of 120 min (AUEC_0→120 min_) of the rat tail flick test curve was calculated using the linear trapezoidal rule. The efficacy factor in local anesthetic effects of bupivacaine after topical application of bupivacaine gel containing polyoxyethylene 2-oleyl ether was compared with the control gel devoid of any additives (14). The efficacy factor (EfF) was calculated using the following equation:

EfF = (AUEC of bupivacaine gels containing enhancer) / (AUEC of the control gel)

## Results and Discussion

*Effects of HPMC concentration on bioadhesive forces and viscosity*

**Figure 1:**
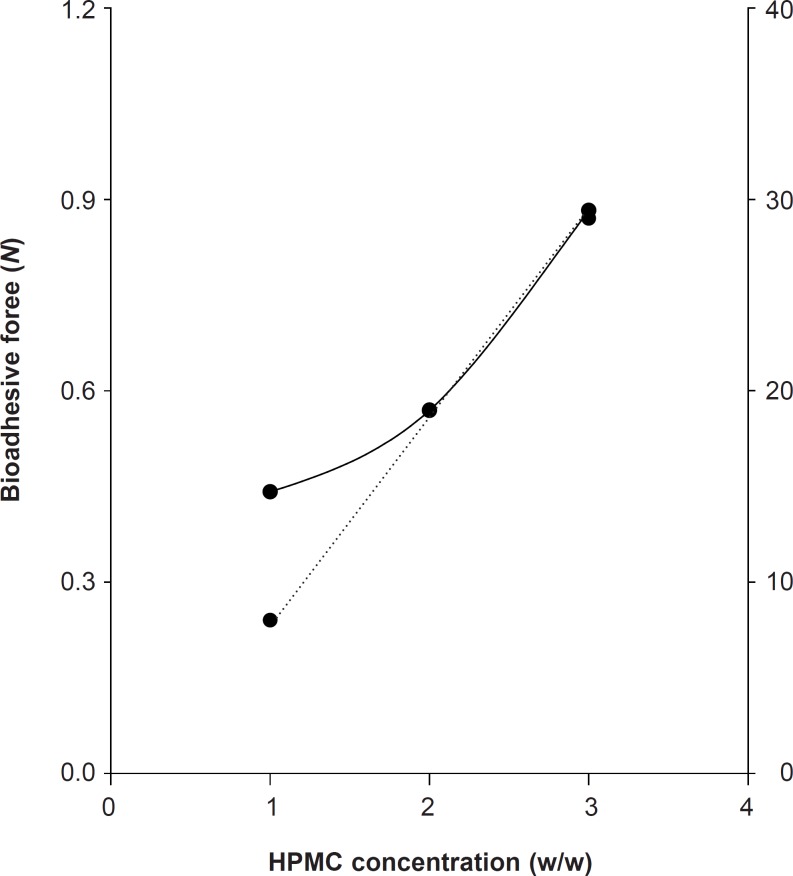
Bioadhesive forces and viscosity of HPMC-K100M at 2% -●-  bioadhesive forces, -●- viscosity

To formulate the bioadhesive gels, we compared the viscosity and bioadhesive forces of HPMC. The viscosity of HPMC-K100M gels at 1%, 2% and 3% was 8 Pa·s, 19 Pa·s, and 29 Pa·s, respectively. The bioadhesive force of HPMC-K100M at 1%, 2%, and 3% was 0.4413, 0.5688, and 0.8826 N, respectively. The increase in the viscosity of HPMC gels led to the increase in bioadhesive forces (Figure 1).

To develop the new gel formulation that has suitable bioadhesion, the easy determination of viscosity could be used instead of bioadhesive force assessment.

*Effect of bupivacaine concentration on drug release*

**Figure 2:**
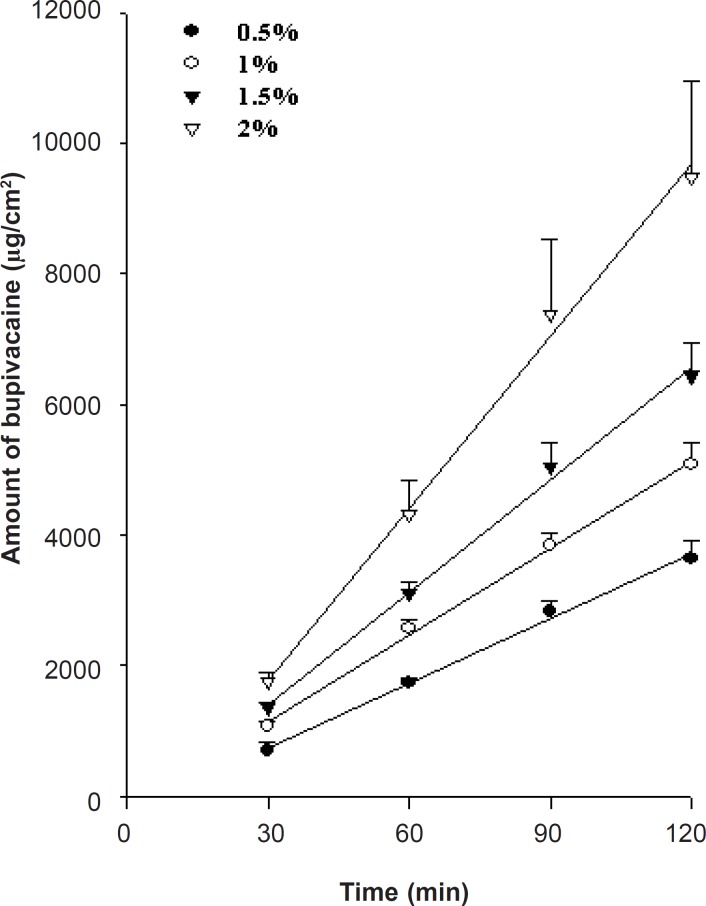
Effect of bupivacaine concentration on drug release from the HPMC gels

To formulate the appropriate bupivacaine concentration, drug release from the prepared HPMC gels across a synthetic cellulose membrane (Spectra/Por, MW 12-14,000) was studied at 37 ± 0.5°C, in amount of 0.5, 1, 1.5 and 2%. Higher bupivacaine concentrations showed faster release (Figure 2) and followed Fick’s law of concentration-dependent passive diffusion. Regarding the commercial product, the bupivacaine concentration was formulated at 1%.

*Effect of enhancers on the permeation of bupivacaine across the rat skin*

To increase the skin permeation of bupivacaine from the HPMC gels, each enhancer such as the saturated and unsaturated fatty acids, pyrrolidones, propylene glycol derivatives, glycerides, and non-ionic surfactants was incorporated in the bupivacaine-HPMC gels (14). Skin is a complex, dynamic layer organ that has many functions beyond its role as a barrier to the environment. The highly organized structure of the stratum corneum (SC) forms a barrier to substance the penetration. Penetration enhancers, accelerants or promoters can interact with some components of skin to increase the fluidity in the intercellular lipid lamellae, the SC to swell, and/or leach out structural components and thus increase the drug penetration through the barrier membrane (15-17).

Fatty acids are capable of being inserted between the hydrophobic tails of the stratum corneum lipid bilayer, disturbing their packing, increasing their fluidity, and subsequently decreasing the diffusion resistance to permeates. When introduced into the predominantly saturated, straight-chained lipid environment of the SC, these kinked fatty acids intercalate and disrupt the ordered lipid array (18) and form separate fluid states that disorder the endogenous lipids (19).

Surfactants enhance the permeability of drugs (20, 21). Pre-treatment with a non-ionic surfactant showed that the SC was loosely layered and that intercellular spaces were wide (21). Brij 92 (polyoxyethylene 2-oleyl ether) produced the best enhancement of bupivacaine gels of the non-ionic surfactants (Table 1). Propylene glycol (PG) is widely used as a vehicle for penetration enhancers and permeates well through the human stratum corneum. PG readily permeates the skin and may carry the drug molecules across (22). The permeation of PG through the tissue could alter thermodynamic activity of the drug in the vehicle, which would in turn modify the driving force for the diffusion (23).

Pyrrolidones have been used as penetration enhancers in human skin for hydrophilic and lipophilic permeants. In terms of mechanisms of action, the pyrrolidones partition well into human corneum stratum. Within the tissue, the pyrrolidones may alter the nature of the membrane and generate ‘reservoirs’ within the skin membranes. Such a reservoir effect, offers potential for sustained release of a permeant from the stratum corneum over the extended time periods (24).

Among the tested enhancers such as the saturated and unsaturated fatty acids, the pyrrolidones, the propylene glycol derivatives, the glycerides, and the non-ionic surfactants, polyoxyethylene 2-oleyl ether showed the highest enhancement. Enhancement factor of the dibucaine gels containing polyoxyethylene 2-oleyl ether was 2.05 in comparison with the dibucaine gels containing the no-enhancer.

*Tail-flick test of bupivacaine gel containing enhancer and vasoconstrictor*

**Figure 3:**
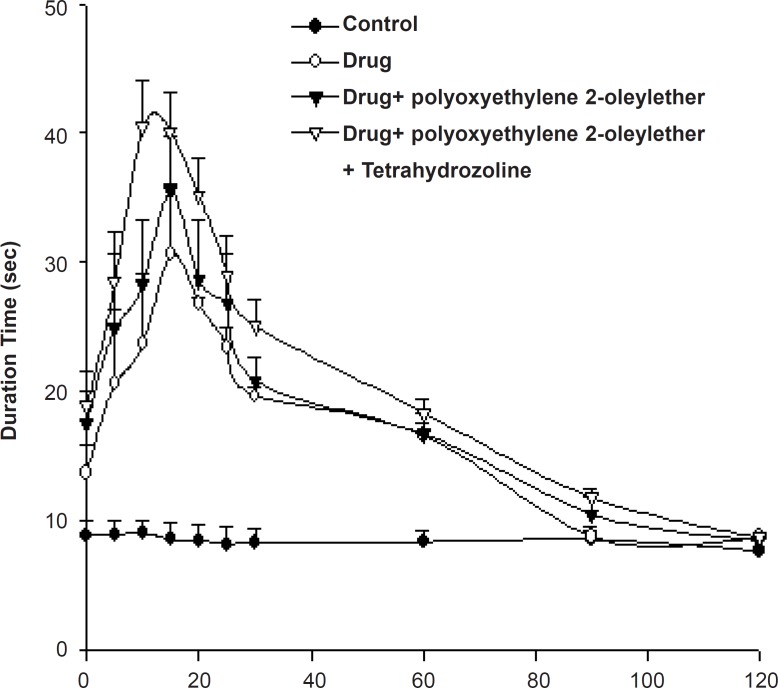
Tail flick test of 1% bupivacaine gels (mean ± SD, n = 3).

In percutaneous permeation studies, bupivacaine gels containing polyoxyethylene 2-oleyl ether showed the best enhancing effects. Therefore, we tested the anesthetic effects of the formulated gel preparation using a rat tail flick analgesic meter (Figure 3).

Table 2 shows the AUEC_0→120min_ of the rat-tail flick test for bupivacaine gels. The AUEC value of bupivacaine gel containing polyoxyethylene 2-oleyl ether was 2073 ± 203 sec/min, while that of enhancer-less gels and polyoxyethylene 2-oleyl ether-container and tetrahydrozoline-container ones were 1894 ± 92 and 2385 ± 73 sec/min, respectively.

**Table 1:** Enhancement factor of enhancers

**Enhancer**	**Flux (μg/cm** ^2^ **/h)**	**EF**
Control	0.12 ± 0.03	1.00
polyoxyethylene 23-lauryl ether	0.14 ± 0.04	1.17
polyoxyethylene 2-stearyl ether	0.20 ± 0.03	1.67
polyoxyethylene 2-oleyl ether	0.15 ± 0.06	1.25
oleic acid	0.13 ± 0.04	1.10
linoleic acid	0.15 ± 0.03	1.25
caprylic acid	0.13 ± 0.03	1.10
lauric acid	0.13 ± 0.04	1.10
myristic acid	0.14 ± 0.13	1.17
oleoyl macrogol-6 glycerides	0.33 ± 0.04	2.75
caprylocaproyl macrogol-8 glycerides	0.18 ± 0.06	1.50
propylene glycol mono caprylate	0.18 ± 0.09	1.50
propylene glycol laurate	0.14 ± 0.09	1.17
propylene glycol monolaurate	0.20 ± 1.01	1.67
NMP	0.35 ± 0.04	2.91
2-pyrrolidone	0.13 ± 0.09	1.10
PVP	0.20 ± 0.06	1.67

According to the rat tail flick test, the local anesthetic efficacy of bupivacaine gel containing polyoxyethylene 2-oleyl ether showed about 2.06-fold compared with that of the gel without polyoxyethylene 2-oleyl ether. The bupivacaine gels containing polyoxyethylene 2-oleyl ether showed the highest anesthetic effects at the 15^th^ min, while bupivacaine gels without enhancer showed anesthetic effects at the 18^th^ min. The bupivacaine gel containing polyoxyethylene 2-oleyl ether showed the fast, prolonged anesthetic effects.

**Table 2:** The comparison of AUeC_0→120min_ for the bupivacaine gel containing an enhancer or not from the rat tail flick test

	**AUeC (sec/min)**	**Efficacy factor**
Control	1008.8 ± 107.4	1
Bupivacaine gel	1894.7 ± 92.6	1.87
Bupivacaine gel containing polyoxyethylene 2-oleyl ether	2073.1 ± 203.0	2.06
Bupivacaine gel containing polyoxyethylene 2-oleyl ether and THZ	2385.9 ± 73.7	2.36

The bupivacaine gel containing polyoxyethylene 2-oleyl ether and tetrahydrozoline showed a 2.36-fold increase in anesthetic activity compared to the control gel without any additives. From the study of the effects of vasoconstrictor on anesthetic effects, bupivacaine gel containing polyoxyethylene 2-oleyl ether and tetrahydrozoline showed more prolonged anesthetic effect compared with that of the tetrahydrozoline-less one (Figure 3).

## Conclusions

As the viscosity of the HPMC gels was increased, the bioadhesive forces were increased as well. To develop the new gel formulation that has suitable bioadhesion, the easy determination of viscosity could be used instead of the bioadhesive force assessment. The rate of drug release was increased with increasing the drug concentration. Among the enhancers, polyoxyethylene 2-oleyl ether showed the highest enhancing effects on drug permeation through the skin. In the rat tail flick test, the area under the efficacy curve of bupivacaine gel containing polyoxyethylene 2-oleyl ether and tetrahydrozoline showed a 2.36-fold increase in anesthetic activity compared to the control gel without any additives. These results suggest that a topical gel formulation of bupivacaine containing enhancer and vasoconstrictor could be developed for enhanced local anesthetic action.
